# Mitochondrial Dysfunction in Arrhythmia and Cardiac Hypertrophy

**DOI:** 10.31083/j.rcm2412364

**Published:** 2023-12-25

**Authors:** Xiaomei Wang, Qianxue Yu, Xuemei Liao, Mengying Fan, Xibin Liu, Qian Liu, Manru Wang, Xinyu Wu, Chun-Kai Huang, Rubin Tan, Jinxiang Yuan

**Affiliations:** ^1^College of Basic Medical, Jining Medical University, 272067 Jining, Shandong, China; ^2^Collaborative Innovation Center for Birth Defect Research and Transformation of Shandong Province, Jining Medical University, 272067 Jining, Shandong, China; ^3^College of Second Clinical Medicine, Jining Medical University, 272067 Jining, Shandong, China; ^4^Department of Cardiology, Ruijin Hospital, Shanghai Jiao Tong University, School of Medicine, 200025 Shanghai, China; ^5^College of Basic Medical, Xuzhou Medical University, 221004 Xuzhou, Jiangsu, China; ^6^Lin He's Academician Workstation of New Medicine and Clinical Translation, Jining Medical University, 272067 Jining, Shandong, China

**Keywords:** mitochondria, dysfunction, heart, arrhythmias, cardiac hypertrophy

## Abstract

Arrhythmia and cardiac hypertrophy are two very common cardiovascular diseases that can lead to heart failure and even sudden death, thus presenting a serious threat to human life and health. According to global statistics, nearly one million people per year die from arrhythmia, cardiac hypertrophy and other associated cardiovascular diseases. Hence, there is an urgent need to find new treatment targets and to develop new intervention measures. Recently, mitochondrial dysfunction has been examined in relation to heart disease with a view to lowering the incidence of arrhythmia and cardiac hypertrophy. The heart is the body’s largest energy consuming organ, turning over about 20 kg of adenosine triphosphate (ATP) per day in the mitochondria. Mitochondrial oxidative phosphorylation (OXPHOS) produces up to 90% of the ATP needed by cardiac muscle cells for contraction and relaxation. Dysfunction of heart mitochondria can therefore induce arrhythmia, cardiac hypertrophy and other cardiovascular diseases. Mitochondrial *DNA *(*mtDNA*) mutations cause disorders in OXPHOS and defects in the synthesis of muscle contraction proteins. These lead to insufficient production of secondary ATP, increased metabolic requirements for ATP by the myocardium, and the accumulation of reactive oxygen species (ROS). The resulting damage to myocardial cells eventually induces arrhythmia and cardiac hypertrophy. Mitochondrial damage decreases the efficiency of energy production, which further increases the production of ROS. The accumulation of ROS causes mitochondrial damage and eventually leads to a vicious cycle of mitochondrial damage and low efficiency of mitochondrial energy production. In this review, the mechanism underlying the development of arrhythmia and cardiac hypertrophy is described in relation to mitochondrial energy supply, oxidative stress, *mtDNA* mutation and Mitochondrial dynamics. Targeted therapy for arrhythmia and cardiac hypertrophy induced by mitochondrial dysfunction is also discussed in terms of its potential clinical value. These strategies should improve our understanding of mitochondrial biology and the pathogenesis of arrhythmia and cardiac hypertrophy. They may also identify novel strategies for targeting mitochondria in the treatment of these diseases.

## 1. Introduction

Mitochondria play a key role in the normal functioning of the heart and in the pathogenesis of various heart diseases [1]. They are multifunctional organelles with a double membrane structure and are ubiquitous in eukaryotic cells. Mitochondria have four functional areas: the inner mitochondrial membrane, outer mitochondrial membrane, intermembrane mitochondrial space (IMS), and membrane matrix. These are the sites for biological oxidation in the mitochondrial electron transport chain (ETC) and oxidative phosphorylation (OXPHOS) [2]. Mitochondria are the major energy-supplying organelle in eukaryotic cells. Adenosine triphosphate (ATP) is produced *in vivo* by three pathways: the tricarboxylic acid cycle, electron exchange in the ETC, and OXPHOS. The mitochondrial OXPHOS system supplies approximately 90% of the energy in eukaryotic cells by producing and releasing ROS, which play an important role in signal transduction to the cytoplasm [3]. Recent studies have shown that mitochondria produce excessive ROS in response to certain stimuli, thereby affecting the normal function of cardiomyocytes involving ion channels and related proteins. Under certain stimuli, the oxidation-reduction balance is disrupted and excessive cellular production of ROS leads to oxidative stress. Both excessive ROS production and oxidative stress are linked to heart diseases, including cardiac hypertrophy and heart failure (HF) [4, 5].

Loss of OXPHOS means that mitochondria are unable to provide energy to tissues and organs with high energy demands such as the heart and brain, leading to related diseases [6]. The IMS is a water chamber located between the inner and outer membranes of the mitochondria and contains cytochrome-c (Cyt-c), adenosine diphosphate/ATP-converting proteins, biological factors, and some enzymes. In addition to being involved in protein and lipid exchange between the matrix and cytoplasm, the IMS is involved in regulating respiratory, metabolic, and apoptotic signals, as well as mitochondrial dynamics. Mitochondria also have important roles in controlling *in vivo *homeostasis of calcium ion (${\mathrm{Ca}}^{2+}$) concentrations [7], ${\mathrm{Ca}}^{2+}$ signal transduction during apoptosis [8], membrane potential, and programmed cell death [9].

A single continuous space in the mitochondrial matrix containing reactive enzymes and mitochondrial *DNA* (*mtDNA*) is involved in biological transformation and synthesis. In mammals, *mtDNA* encodes 37 genes, comprising two *rRNA* genes (*12S* and *16S*), *22 tRNA* genes, and 13 genes encoding mitochondrial ETC and OXPHOS-related protein subunits [10]. Many studies have shown that abnormal copies and mutations in *mtDNA* may be associated with various heart diseases and can lead to dysfunctional OXPHOS and disease [11]. Mitochondrial dynamics includes two major processes: mitochondrial fusion and division. Mitosin 1 (Mfn1), mitosin 2 (Mfn2) and optical atrophy protein 1 (OPA1) participate in mitochondrial fusion. Gtpase kinetics related protein (DRP1), mitochondrial fission protein 1 (FIS1) and mitochondrial fission factor (MFF) participate in mitochondrial division [12]. Mitochondria are considered to be the key sensors and effectors of cardiac pathophysiology. In addition to the ability to produce energy, cardiac mitochondria also directly regulate several other intracellular processes, such as calcium homeostasis, apoptosis, nuclear gene expression, ion gradient, cell redox potential and contractility. Balancing mitochondrial fission/fusion is essential for these functions [13, 14, 15, 16, 17]. An imbalance in mitochondrial dynamics can easily induce the occurrence of heart-related diseases, such as cardiac hypertrophy and heart failure [18, 19].

Arrhythmias and cardiac hypertrophy are mitochondrial diseases. Mitochondria form the core of excitation-contraction coupling in cardiomyocytes [20]. The opening of voltage-gated L-type ${\mathrm{Ca}}^{2+}$ channels at the plasma membrane allows an influx of extracellular ${\mathrm{Ca}}^{2+}$ and activates the ryanodine receptor 2 (RyR2) in the sarcoplasmic reticulum (SR), which then releases ${\mathrm{Ca}}^{2+}$ from the SR into the cytoplasm. Myocardial contraction occurs when free cytosolic ${\mathrm{Ca}}^{2+}$ binds to troponin [21]. The activity of RyR2 is positively correlated with ATP concentration [22]. At the end of myocardial contraction, sarcoplasmic/endoplasmic reticulum ${\mathrm{Ca}}^{2+}$ATPase 2 allows reentry of ${\mathrm{Ca}}^{2+}$, while ${\mathrm{Na}}^{+}$/${\mathrm{Ca}}^{2+}$ exchange across the plasma membrane, exporting intracellular ${\mathrm{Ca}}^{2+}$ to relax the myocardium [22, 23]. This process is dependent on the mitochondrial energy supply. Mitochondria take up excess ${\mathrm{Ca}}^{2+}$ during cardiomyocyte overload to reduce the burden on the heart. Mitochondria also regulate intracellular ${\mathrm{Na}}^{+}$ and $K^{+}$ homeostasis, thereby preventing adverse effects of these ions on cardiac contractility [24], providing energy for cardiomyocyte excitation-contraction coupling, affecting cardiac diseases such as cardiac hypertrophy and arrhythmias. In recent years, mitochondrial dysfunction has increasingly been linked to heart-related diseases. In this review, we summarize the recent literature on the relationship between mitochondrial dysfunction and arrhythmia and cardiac hypertrophy. Specifically, we will examine the effects of mitochondrial energy production, oxidative stress, and *mtDNA* mutations on these two diseases. We also review evidence obtained using human and animal models, and explore new therapeutic strategies that could restore mitochondrial function and human health.

## 2. Mitochondria and Arrhythmias

Arrhythmia refers to the abnormal conduction, frequency, rhythm, and origin of electrical impulses in the heart. The clinical manifestations of this disease are a rapid, slow, or irregular pulse rhythm, chest tightness, palpitation, dizziness, and suffocating wheezing. Pathogenic factors in arrhythmia include HF, aging, overweight/obesity, and inflammation [25]. Diabetic heart disease, cardiac dysfunction, and hereditary diseases are also associated with arrhythmia [22]. Pathophysiological changes associated with arrhythmia include cardiac fibrosis, mechanical stress-induced ventricular refractory period changes, and electrophysiological changes in Purkinje fibers. The global prevalence of atrial fibrillation (AF) is approximately 0.51% [26]. Out-of-hospital sudden cardiac death is responsible for >60% of deaths due to cardiovascular diseases [27]. Various types of arrhythmias, such as AF, ventricular fibrillation, and extrasystole [28] can lead to a variety of complications that pose a risk for the progression of heart disease. These can result in decreased cardiac function or even the development of HF, thus seriously endangering human health. A better understanding of the mechanisms that underlie arrhythmia is paramount to addressing this problem. Mitochondria are the key factor related to arrhythmia, and the intrinsic relationships between mitochondrial energy disorder, oxidative stress, *mtDNA* mutation, and arrhythmia will be elaborated below.

### 2.1 Mitochondrial Energy Disorders and Arrhythmia

Samuel *et al*. [29] found that ATP depletion increased the risk of arrhythmias in a study of 46 patients with low left ventricular ejection fraction. Emelyanova *et al*. [30] found that the activity of ATP-producing multi-subunit complexes in the mitochondria of cardiac tissue from patients with AF was lower than in healthy individuals. Tu *et al*. [31] quantitatively compared key enzymes related to mitochondrial energy metabolism in the left atrial appendage of patients with permanent AF valvular disease. Studies in diabetic patients with AF have shown the presence of damaged complexes in the mitochondrial respiratory chain, increased oxidative damage, and decreased ATP production [32]. Moreover, several genes related to mitochondrial oxidative phosphorylation are down-regulated in patients with postoperative AF [33]. Hou *et al*. [34] found a mutation in the trans-2, 3-enoyl-CoA reductase-like (*TECRL*) gene in a patient with catecholaminergic polymorphic ventricular tachycardia (CPVT). Moreover, a *TECRL*-null mouse model showed significant cardiac dysfunction, while electron microscopy showed that the mitochondria in the myocardium of these mice were irregularly arranged and the cristae were missing. The expression of proteins involved in mitochondrial OXPHOS and ATP production were also significantly reduced. Mitochondrial respiration was measured using human-induced pluripotent stem cells and H9C2 cells. The results showed that overexpression of *TECRL* enhanced mitochondrial respiration through phosphoinositide-3-kinase/murine thymoma viral oncogene homolog signaling. In contrast, cardiomyocytes from *TECRL*-null mice showed increased expression of mitofusin2, decreased expression of p-Akt (Ser473) and nuclear factor erythroid 2-related factor 2, and increased expression of apoptosis inducing factor and Cyt-c [35]. This resulted in reduced mitochondrial ATP production, which was one of the causes of CPVT [34].

### 2.2 Mitochondrial Oxidative Stress and Arrhythmia

In addition to producing ATP, mitochondria also produce ROS as a by-product of OXPHOS. Under physiological conditions, trace ROS establish a mitochondria-driven signaling network that integrates metabolism with gene transcription and enzyme activity [10, 11]. A short-term increase in ROS signaling triggers an adaptive response and promotes preconditioning, thereby increasing the resistance of cells and tissues to injury [20, 21]. Studies have shown that excessive ROS levels can lead to changes in cell function and increased cell death [25]. Electric shock defibrillation is a major source of ROS during the treatment of patients with cardiac arrest. Using a canine model of defibrillation shock, Caterine *et al*. [36] demonstrated that shock energy was positively correlated with the ascorbate free radical peak. Tsai *et al*. [37] created a rat model of cardiac arrest due to ventricular fibrillation. Animals were defibrillated and then grouped according to temperature and to the use of ascorbic acid (AA), an antioxidant. The Malondialdehyde (MDA) -586 method was then used to identify oxidative damage in the myocardium, with the results showing that damage in all groups was significantly increased. The group with normal body temperature and AA showed rapid heart rate recovery and improved systolic function and survival rate, while the group with low body temperature and AA showed improved systolic and diastolic functions [37]. Yoo *et al*. [38] found that mitochondrial ROS and NADPH oxidase 2 activity were higher in the atrial tissues of dogs with rapid atrial pacing (RAP) than in controls. Acetylcholine-dependent K current (IKH) is a frequency-related ion channel that can promote shortening of the effective refractory period in AF and induce arrhythmia. In the same study by Yoo *et al*. [38], the IKH cell density decreased significantly after RAP muscle cells were treated with ROS inhibitors. In addition to the promoting effect of ROS, the interaction between ${\mathrm{Ca}}^{2+}$ and ROS is another cause of arrhythmia [38]. A tight interaction exists between the endoplasmic reticulum (ER)/SR and mitochondria, whereby the inositol 1, 4, 5-triphosphate/RyR2 receptor allows ${\mathrm{Ca}}^{2+}$ transport from the ER/SR to the mitochondria [39]. Crosstalk between mitochondria and the SR regulates ${\mathrm{Ca}}^{2+}$ transport and matches energy supply and demand by regulating mitochondrial respiration [40]. This mechanism has also been implicated in arrhythmias [41]. For example, mitochondrial ROS emission is increased by excessive RyR2 and SR ${\mathrm{Ca}}^{2+}$ leakage [42]. Hamilton *et al*. [43] synthesized adenoviruses carrying biosensor constructs and sub-cultured ventricular myocytes (VMs) from CPVT mice. The mitochondrial-specific ROS indicator, MitoSOX, showed a significant increase in ROS emissions in CPVT VMs compared with the control group. Western blotting also showed a significant increase in the oxidative state of RyR2 immunoprecipitated from diseased VMs. Exciting the intracellular RyR2 receptor with low doses of caffeine sharply increased SR ${\mathrm{Ca}}^{2+}$ leakage, while the influx and efflux of ${\mathrm{Ca}}^{2+}$ into and out of the mitochondrial matrix also increased. This phenomenon improved after treatment with mito-TEMPO, a ROS inhibitor. Overexpression of the dominant-negative mitochondrial ${\mathrm{Ca}}^{2+}$ uniporter inhibited the uptake of mitochondrial ${\mathrm{Ca}}^{2+}$ and the release of mitochondrial ROS. The study by Hamilton *et al*. [43] also showed that an overactive RyR2 channel increases SR ${\mathrm{Ca}}^{2+}$ leakage. Following mitochondrial ${\mathrm{Ca}}^{2+}$ uptake, mitochondrial-derived ROS further drives SR ${\mathrm{Ca}}^{2+}$ leakage to form a positive feedback process. Eventually, excessive ${\mathrm{Ca}}^{2+}$ in cardiac VMs breaks the balance and drives CPVT [43].

### 2.3 MtDNA and Arrhythmia

*MtDNA *mutations may be associated with the development of cardiovascular diseases, and cardiovascular involvement is very common in patients with pathogenic *mtDNA*. Dysfunction of *mtDNA* has been associated with an increased risk of AF [44]. The *mtDNA* copy number in humans is proportional to mitochondrial gene transcription, as well as being a marker of mitochondrial dysfunction and being negatively correlated with the risk of AF. Therefore, a low *mtDNA* copy number is considered to be a key factor in AF [44], with the *mtDNA 4977 *mutation being the main cause. Lin *et al*. [45] screened for large-scale deletions of *mtDNA* in the atrial muscle of patients with AF. The *4977* bp deletion was the most frequent and abundant, with the incidence of this deletion being higher in patients with AF than in those without [45]. These workers also performed quantitative polymerase chain reaction to evaluate *mtDNA* lesions caused by oxidative damage. The extent of *mtDNA* damage in patients with AF was found to be greater than in patients without AF. A study of *mtDNA *changes in four patients with chronic AF and two matched patients without chronic AF found mutations only in the *mtDNA* control region and coding region of the patients with chronic AF [46]. These results demonstrate the key role of *mtDNA* mutation in arrhythmia. Atrial fibrosis, another major cause of arrhythmia, is considered to be a marker of AF-related structural remodeling and the cause of persistent AF [47]. Therefore, *mtDNA* damage is involved in the pathological mechanism of arrhythmia.

### 2.4 Mitochondrial Dynamics and Arrhythmia

Altered mitochondrial dynamics are associated with the development of cardiac arrhythmias. Specifically, mutations in the Emerin protein, which is encoded by the human Emerin (*EMD*) gene, have been linked to Emery-Dreifuss muscular dystrophy type 1. Recent research by Du *et al*. [48] discovered a novel mutant variant present in patients afflicted with this disorder, unveiling a dual impact characterized by muscle weakness and the emergence of arrhythmias. Subsequent *in vitro* experiments have further confirmed that silencing the *EMD* gene or attenuating the expression of its encoded protein precipitates a notable reduction in key genes such as MFN and Dynamin-related protein 1 (DRP1), ultimately disturbing the intracellular mitochondrial network [48]. Arrhythmia stands as a frequent complication arising from ischemia-reperfusion in the heart [49]. In a study by Lahnwong *et al*. [50], male rats were administered the sodium-glucose cotransporter protein 2 inhibitor, dapagliflozin, prior to ischemia. The administration of dapagliflozin before ischemia was found to decrease the size of cardiac infarcts, lower parameters associated with arrhythmia, reduce cardiomyocyte apoptosis, and elevate the levels of OPA1 in the cardiac tissues of the rats [50]. Moreover, researchers have observed that rats fed on a high-fat diet exhibit an increased incidence of arrhythmia and mortality compared to those fed on a normal diet [51]. Chen *et al*. [52] discovered that male rats fed on a high-fat diet displayed fat deposition and cardiac insufficiency. These rats also exhibited decreased mitochondrial density and abnormal morphology, along with significantly reduced protein expression levels of the genes encoding Mfn1, Mfn2, and OPA1. Conversely, the protein expression levels of DRP1 and FIS1 genes were significantly up-regulated [52].

In a study by Murphy *et al*. [53], using a primary rabbit left ventricular cardiomyocyte model, it was found that the increased probability of arrhythmia in elderly rabbits was due to an increase in the expression level of DRP1 in cardiac tissues, rather than Mfn2, as there were no changes in the expression level of Mfn2 [53]. Pathophysiological changes stemming from HF have been closely correlated with the development of ventricular arrhythmias [54]. Mouse heart models with specific knockdowns of Mfn1 and Mfn2 genes presented excessive mitochondrial fragmentation within the myocardium, coupled with aberrant respiratory chain function, culminating in lethal HF [55]. Notably, altered Drp1 has been identified as a plausible causative factor in the pathogenesis of dilated cardiomyopathy, indicating that genetic variations within the Drp1 gene might play a pivotal role in dilated cardiomyopathy and potentially be associated with arrhythmias as well [56]. Specific knockdown of the cardiac Drp1 gene gave rise to diminished myocardial mitochondrial autophagy, an escalation in the population of dysfunctional mitochondria, and the gradual progression of left ventricular dysfunction in mice [57]. In addition, *in vitro* and *in vivo* experiments elucidated that the inhibition of Drp1 expression by midivi exhibited the capacity to enhance mitochondrial membrane potential, reduce excessive mitochondrial division, and suppress cell apoptosis [58, 59], thereby yielding a protective effect against arrhythmias. The orchestration of mitochondrial hyperfusion and arrhythmia has been notably linked to Mfn2, as unveiled by Ishaq *et al*. [60]. Through *in vitro* studies utilizing HL-1 cardiomyocytes, it was demonstrated that Mfn2 played a pivotal role in coordinating mitochondrial binding to SR and facilitating mitochondria-associated memberanes(MAM)formation, thereby propelling mitochondrial hyperfusion and impacting arrhythmogenesis. Another factor that may contribute to the occurrence of arrhythmias [61] and mitochondrial damage [62] is PM2.5, a particulate matter constituting air pollution. Research has underscored the deleterious impact of PM2.5 exposure on mitochondrial function, substantiated by the down-regulation of the mitochondrial fusion gene Mfn1, concurrent up-regulation of mitochondrial autophagy genes (*PINK1* and *Parkin*), and an escalation in the expression of mitochondrial fragmentation genes (*MFF* and *FIS1*). These molecular changes have been closely correlated with larger infarct size in the hearts of rats with myocardial infarction, disruption of normal sinus rhythm, ventricular arrhythmias, and mitochondrial damage [63]. In a diabetic rat model, Shao *et al*. [64] conducted gene expression analyses, unveiling diminished protein expression levels of mitochondrial fission and fusion proteins, with the exception of OPA-1, DRP-1, and Mfn1. This dysregulation of mitochondrial dynamics resulted in the inhibition of mitochondrial fusion and fission, which subsequently contributed to left heart stomatal fibrosis and a marked escalation in the incidence of atrial fibrillation [64].

The tightly regulated realm of mitochondrial autophagy within cells emerges as a highly precise and strict process. Imbalances in mitochondrial autophagy, leading to the accumulation of senescent, impaired, or dysfunctional mitochondria within the cellular milieu, inevitably curtails energy provision and potentially triggers apoptosis. In instances where mitochondrial autophagy experiences impairment within cardiac tissue cells, ATP production wanes, subsequently influencing the functionality of ion channels and transport proteins situated in the sarcoplasmic membrane and SR—entities heavily reliant on ATP [28]. The repercussions resonate in affecting the heart’s action potential, potentially serving as an instigator of arrhythmias. Noteworthy is the perturbation of mitochondrial autophagy, subsequently inducing aggregation and necrosis of compromised mitochondria, thereby unleashing an influx of ROS [65]. This heightened ROS presence disrupts intracellular redox equilibrium, perpetuating the prolongation of Early Afterdepolarization, Delayed Afterdepolarization, and action potentials [66]. Elevated ROS levels further impact the modulation of ion channel protein expression levels, such as ${\mathrm{Na}}^{+}$ or ${\mathrm{Ca}}^{2+}$ [43, 67, 68], while also regulating entities like RyR2 and calmodulin-dependent protein kinase II [69, 70]. This intricate interplay reverberates throughout cardiac potential, thus contributing substantively to the development of arrhythmias.

**Fig. 1.**
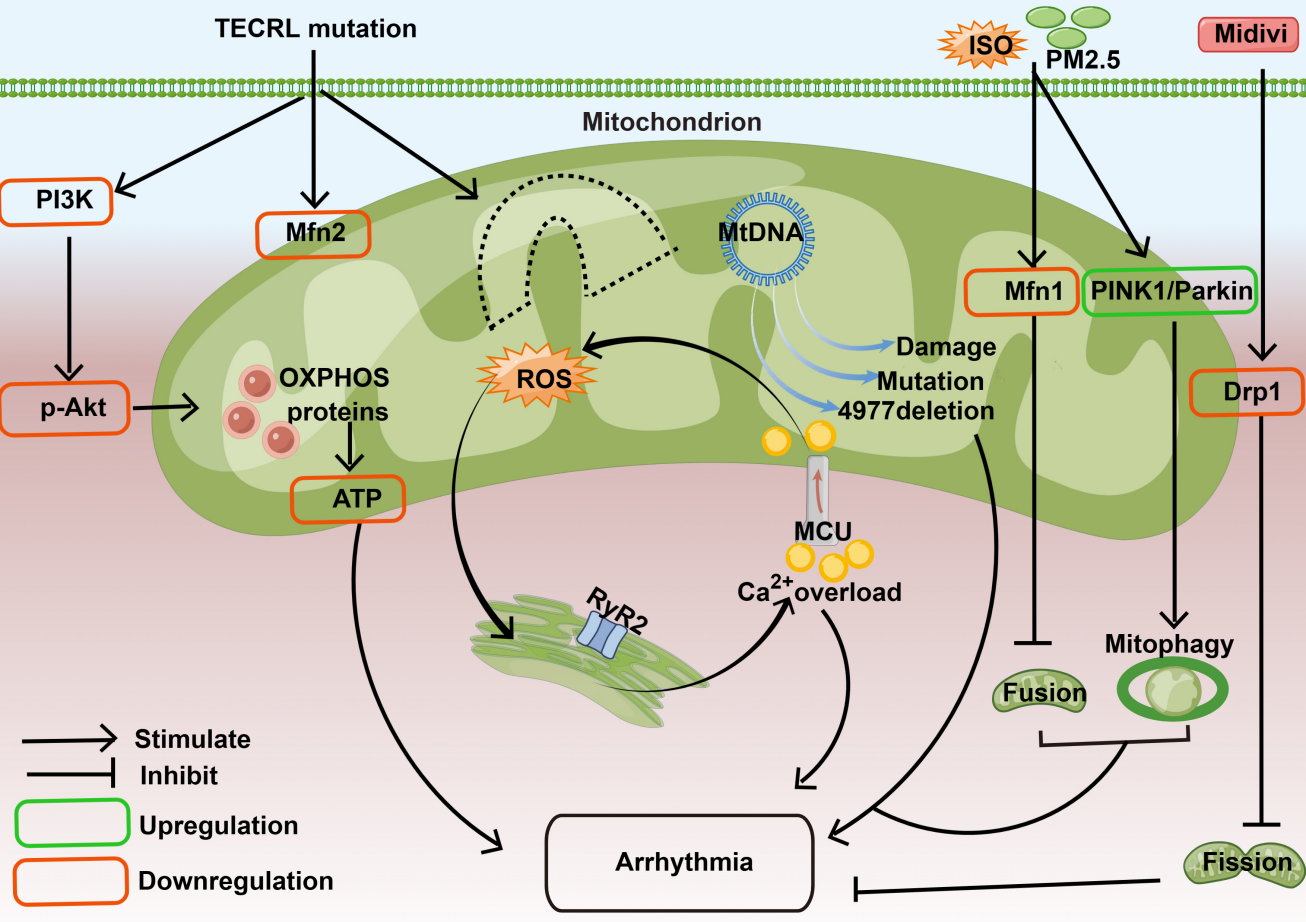
**Mechanisms by which mitochondria are involved in regulating cardiac arrhythmias through energy control, oxidative stress, mitochondrial *DNA*, mitochondrial dynamics, and more**: In the mouse *TECRL* KO model, the PI3K/AKT pathway is inhibited in cardiomyocytes, the expression of mitochondrial Mfn2 protein is decreased, the expression of OXPHOS-related proteins is decreased, ATP production is decreased, and arrhythmia is induced. In an *in vitro* culture model of ventricular myocytes isolated from CPVT mice, we detected excessive RyR2 channels and increased SR ${\mathrm{Ca}}^{2+}$ leakage. After excessive ${\mathrm{Ca}}^{2+}$in the cytoplasm was taken up by MCU on the mitochondria, mitochondrial ${\mathrm{Ca}}^{2+}$ overload was induced, which led to increased ROS production and further driven SR ${\mathrm{Ca}}^{2+}$ leakage and formed a positive feedback process. It drives the onset of arrhythmias. Mitochondrial dysfunction caused by *mtDNA* mutations, *mtDNA4977* deficiency and *mtDNA* damage is one of the important causes of arrhythmia. In MI rats exposed to PM2.5 followed by ISO injection, the protein expression of Mfn1 was down-regulated, and the protein expression of PINK1 and Parkin was up-regulated, which aggravated the infarct size, destroyed the normal sinus rhythm, and triggered ventricular arrhythmias. In the HL-1 cell model, Drp1 inhibitor midivi exerted anti-arrhythmia effect by inhibiting the expression of Drp1, enhancing mitochondrial membrane potential, reducing mitochondrial fission and inhibiting cell apoptosis. ATP, adenosine triphosphate; Akt, V-akt murine thymoma viral oncogene homolog; ${\mathrm{Ca}}^{2+}$, calcium ion; CPVT, catecholaminergic polymorphic ventricular tachycardia; Drp1, dynamin-related protein 1; ISO, isoproterenol; MCU, mitochondrial calcium uniporter protein; Mfn1, mitofusin-1; Mfn2, mitofusin-2; *MtDNA*, mitochondrial *DNA*; PI3K, phosphoinositide 3-kinase; PINK1, phosphatase and tensin homolog deleted on chromosome ten-induced putative kinase 1; RyR2, ryanodine receptor 2; SR, sarcoplasmic reticulum; *TECRL* KO, Trans-2,3-Enoyl-CoA Reductase-Like Knockout; OXPHOS, oxidative phosphorylation system; ROS, reactive oxygen species; MI, myocardial infarction; PM2.5, particulate matter 2.5; HL-1, mouse cardiomyocyte cell line.

The proposed mechanism by which mitochondria participate in the regulation of arrhythmia is shown in Fig. 1.

## 3. Mitochondria and Cardiac Hypertrophy 

Cardiac hypertrophy is characterized by increased ventricular muscle mass, especially left ventricular hypertrophy. This enhances cardiac contractility, reduces myocardial oxygen consumption, and maintains cardiac output [71, 72, 73]. Cardiac hypertrophy is usually considered a secondary and compensatory change in the myocardium caused by abnormal pressure or volume load [74], and represents an adaptive response. Moderate cardiac hypertrophy can enhance myocardial contractility, reduce cardiac oxygen consumption, and maintain cardiac output [71, 72, 73]. Physiological cardiac hypertrophy is a reversible process [75]. However, under the continuous action of pathogenic factors it can develop into pathological cardiac hypertrophy and increase myocardial oxygen consumption, cause systolic dysfunction, affect heart pump function, and eventually lead to heart failure and potentially sudden death [71]. Mitochondria are the main energy source for the heart and are closely linked to the energy metabolism and oxidative stress functions of myocardium. Dysfunctional mitochondria play an important role in the development of cardiac hypertrophy [76].

### 3.1 Mitochondrial Energy Disorder and Cardiac Hypertrophy

The energy demand of myocardial cells is continuously high. Mitochondria are the source of 90% of ATP production and ensure the energy demand is met [77, 78]. The OXPHOS system is the final step in the ATP production process and is regulated by many mechanisms, including mitochondrial dynamics [79], mitochondrial protein post-translational modifications (PTM) [80], and signal transducer and activator of transcription 3 (STAT3) [81]. PTM dysfunction has been shown to cause mitochondrial dysfunction in patients with heart disease [82, 83]. Sirtuin 3 (SIRT3), a member of the PTM family, is a nicotinamide adenine dinucleotide-dependent histone deacetylase [84] and is responsible for the intra-mitochondrial deacetylation of lysine residues [85]. SIRT3 has been shown to acetylate mitochondrial proteins, thereby damaging mitochondrial energy metabolism [84]. The expression level of SIRT3 in the human left ventricle is negatively correlated with the degree of heart disease [86]. Boardman *et al*. [86] found that 228 human heart tissue samples with low SIRT3 expression were more prone to moderate or extensive interstitial fibrosis, cardiac hypertrophy, ischemic changes, and infarcted tissues compared to samples with high SIRT3 expression. Koentges *et al*. [87] reported that the heart of *SIRT3-KO* mice suffered from contractile dysfunction and oxidative damage of energy substrates, which became worse with time. This change is probably due to the mitochondrial dysfunction in myocardial cells caused by SIRT3 deletion. Of the 84 hyperacetylated mitochondrial proteins was observed in the *SIRT3-KO* mouse model, including three proteases involved in the tricarboxylic acid cycle and 50 protein enzymes involved in the formation of ETC subunits. Increased protein acetylation impairs the energy supply to cardiomyocytes [87]. STAT3 signaling sensors and activators are key mediators of myocardial cell survival and are able to interact with mitochondrial complexes and promote ATP OXPHOS systems. STAT3 can also bind to *mtDNA *and alter the transcription of NADH dehydrogenase 5/6 and cytochrome B [81]. Studies have also shown that STAT3 promotes cardiomyocyte hypertrophy [88]. Using the H9c2 cardiogenic cell line, Jeong *et al*. [89] reported downregulation of ETC complexes II and III and regulation of STAT3 activation in catecholamine-induced cardiac hypertrophy. Phenylephrine and isoproterenol induced cardiomyocyte hypertrophy in H9c2 cells, which then decreased STAT3 expression and phosphorylation in the mitochondria of H9c2 cells. pS727-STAT3 and dysfunction of mitochondrial complex interaction, as well as decreased expression of the ETC complexes II and III, lead to failure of the OXPHOS system to generate sufficient ATP and induce cardiac hypertrophy [89]. In a rat model of cardiac hypertrophy induced by aortic coarctation, comparative mitochondrial protein omics revealed significant defects in the OXPHOS system of the heart, due mainly to decreased expression of the ETC subunits Ndufa9, Sdhb and COX5b [90].

### 3.2 Oxidative Stress and Cardiac Hypertrophy

Mitochondrial oxidative stress promotes cardiac hypertrophy, fibrosis and apoptosis [91]. Mutations in the myosin regulatory light-chain sarcomere gene result in human familial hypertrophic cardiomyopathy. In mouse cardiomyocytes, overexpression of the human glutamic acid to lysine substitution at position 22 (*E22K*) gene carrying a mutation near the Regulatory light-chain ${\mathrm{Ca}}^{2+}$ binding site leads to ventricular hypertrophy [90].

In a mouse model, the *E22K* mutation results in increased expression of the protein kinase C (PKC)/nuclear factor of activated T cell (NFAT) protein axis, leading to central chamber hypertrophy [92]. Activated PKC and NFAT are known to be related to the sensitization of mitochondrial stress signals. Stress factors such as the depletion of ROS, drugs or *mtDNA* can lead to decreased mitochondrial ${\mathrm{Ca}}^{2+}$ uptake, cytoplasmic ${\mathrm{Ca}}^{2+}$ overload, activation of cellular PKC and nuclear response factors including NFAT and cyclic adenosine monophosphate (cAMP) response element-binding protein, and eventually ventricular hypertrophy [93].

Thioredoxins (Trx) are a group of proteins that maintain a stable redox state in the body. They consist of three major types in sperm cells: cytoplasmic Trx1, mitochondrial Trx2, and Trx3 [94]. Andreadou *et al*. [95] reported that Trx1-ablated mice showed increased oxidative stress and cellular hypertrophy compared to non-transgenic mice .

It has been shown that ${\mathrm{Ca}}^{2+}$ can dislocate Cyt-c from the mitochondrial inner membrane, leading to defective complex III and hence increased ROS production [96]. The mitochondrial calcium uptake 1 (MICU1) protein is located in the mitochondrial inner membrane and is responsible for regulating ${\mathrm{Ca}}^{2+}$ uptake. MICU1 sets a threshold during mitochondrial ${\mathrm{Ca}}^{2+}$ uptake to prevent ${\mathrm{Ca}}^{2+}$ overload and stress [97]. Using a leptin receptor-deficient mouse model, Ji *et al*. [98] found that MICU1 was downregulated, and the left ventricular mass and diameter were increased. Moreover, injection of adenovirus expressing MICU1 significantly reduced ROS production in the heart of these mice and alleviated cardiac hypertrophy [98].

Using angiotensin II-induced neonatal mouse VMs, Yang *et al*. [99] showed that knock-down of MICU1 induced mitochondrial membrane potential abnormalities, increased ROS production, and increased the protein and mRNA expression levels of atrial natriuretic peptide, brain natriuretic peptide, and ß-myosin heavy chain (MHC), leading to an increased myocardial cell surface area.

### 3.3 MtDNA and Cardiac Hypertrophy

Several studies have shown that mitochondrial disorders directly promote cardiac hypertrophy [100, 101]. Zhu *et al*. [102] sequenced the complete *mtDNA* genome in a cardiac hypertrophy model of inbred SHRF108 rats and identified 89 *mtDNA* mutations. Of these, 35.5% were in gene-coding sequences, 18.7% were in non-coding *RNA* sequences, and 45.8% were synonymous mutations [102]. *MtDNA* is not protected by histones, thus increasing its susceptibility to oxidative damage and harmful mutations [103]. Cardiac hypertrophy is the most common cardiovascular disease observed in patients with chronic kidney disease (CKD) [104]. Han *et al*. [104] performed *RNA* sequencing of the myocardial expression profile in CKD mice and found upregulation of genes related to ventricular hypertrophy, and downregulation of mitochondrial genes [104]. Cyclic guanosine monophosphate-adenosine monophosphate synthase (cGAS) is a major *DNA *sensor in mammals, and its stimulation activates the adaptor protein stimulator of interferon genes (STING) [105]. Nuclear factor kappa B is the main downstream effector of the cGAS-STING pathway [106]. Mitochondrial oxidative damage in the myocardial cells of CKD mice results in the release of *mtDNA* into the cytoplasm through voltage-dependent anion channel 1-mediated mitochondrial outer membrane permeability, activation of the STING-Nuclear factor kappa B signaling pathway, and initiation of CKD-induced cardiac hypertrophy [104]. VBIT-4, a voltage-dependent anion channel 1-mediated mitochondrial outer membrane permeability inhibitor, reduced the release of *mtDNA* into the cytoplasm, while VBIT-4 treatment was found to significantly reduce CKD-induced cardiomyocyte hypertrophy *in vitro* [104].

In the myocardial cells of mice with diabetic cardiomyopathy, Yan *et al*. [107] found that *mtDNA* released into the cytoplasm following mitochondrial oxidative damage initiated nucleotide-binding oligomerization domain-like receptor pyrin domain containing 3 (NLRP3) inflammasome-dependent cell apoptosis and pro-inflammatory responses through activation of the cGAS-STING signaling pathway, leading to cardiac hypertrophy.

Isoform 1 of uracil-*DNA* glycosylase is the core component of the *mtDNA* repair mechanism and prevents mutations by removing incorrect uracil from *mtDNA * [108]. Mutant uracil-*DNA* glycosylase 1 causes *mtDNA* dysfunction by removing mitochondrial genomic uracil and thymine [108]. α-MHC is heart-specific [109], and in the α-MHC-induced transgenic mutUNG1 mouse model it is specifically expressed in myocardial cells [110]. Lauritzen *et al*. [110] induced the expression of mutUNG1 in mouse cardiomyocytes by oral administration of doxycycline. Compared with wild-type mice, the heart mass and cross-sectional area of cardiomyocytes infected with mutUNG1 were increased, the nuclei of the cardiomyocytes were significantly larger, and the heart was significantly enlarged .

Zidovudine is an antiretroviral drug that interferes with the replication fidelity of *mtDNA* and increases fragment deletion in *mtDNA* [111]. Dai *et al*. [100] reported increased left ventricular weight index and heart weight following treatment of wild-type mice with Zidovudine for 8 weeks, indicating that mice with missing fragments of *mtDNA* develop ventricular hypertrophy.

### 3.4 Mitochondrial Dynamics and Cardiac Hypertrophy

Mitochondrial dynamics indicators from the left ventricle of patients with HF showed significant mitochondrial dynamics abnormalities. Compared with donor samples from normal people, the expression of Mfn2 and OPA-1 in the left ventricular myocardium of patients with heart failure was significantly up-regulated, while the expression of Drp-1 and fission-1 protein was down-regulated [112]. Gene detection of myocardial tissue in patients with hypertrophic cardiomyopathy (HCM) showed that the expression of *hsa-miRNA-20a-5p* in cardiac muscle samples of HCM patients was 2.26 times higher than that of the control group. Further study in AngII-induced rat cardiomyocyte hypertrophy model showed increased *miRNA-20* levels, increased ANP levels, and cardiomyocyte hypertrophy accompanied by decreased Mfn2 protein levels. Target gene prediction programs predicted Mfn2 as a target of *miRNA-20*, indicating that *miRNA-20* may participated in the regulation of the occurrence and development of cardiac hypertrophy by interact with its downstream Mfn2 factor [113]. Another related study utilizing transverse aortic constriction (TAC) induced cardiac hypertrophy model, as well as Ang II-induced HCM model in rats, showed that the expression of *miR-5-17p* in myocardial tissue was up-regulated, and the expression of Mfn2 protein was decreased. The high expression of *miR-17-5p *is involved in the occurrence of ventricular hypertrophy by targeting Mfn2 and inhibiting mitophagy. Further studies showed that in Ang II-induced NRVM, overexpression of Mfn2 could inhibit the Phosphoinositide 3-kinase/Murine thymoma viral oncogene homolog/mechanistic target of rapamycin signaling pathway and enhance cardiomyocyte mitophagy, thereby alleviating the pathological process of cardiac hypertrophy [114]. Similarly, expression of Mfn2 is down-regulated in neonatal rat ventricular cardiomyocytes in a phenylephrine induced hypertrophy model [115].

Evidence from an in *vivo* model confirms the severity of abnormal mitochondrial fusion and division in mice. Combined ablation of Mfn1 and Mfn2 in mice leads to lethal heart failure [55]. Similarly, cardiomyocyte-specific knockout of Drp1 mice died at 6 weeks of age [116].

In the acute pressure overload TAC mice model, a study has shown increased phosphorylation of Drp-1 at S622 in mice left ventricle cardiomyocytes as well as elevated expression of PKC-δ level. Drp-1 was also translocated into the mitochondria after phosphorylation at S622 by PKC-δ. This phosphorylation led to the translocation of Drp-1 into the mitochondria. Furthermore, treatment with chemical inhibitor of Drp-1, mdivi-1, before TAC reduced1 left ventricle hypertrophy induced by pressure overload [117].

Conditional knockout studies targeting Mfn1/Mfn2 and DRP1 have unveiled intriguing findings. Both Mfn1/Mfn2 and DRP1 conditional knockout lead to progressive left ventricular enlargement and decreased ejection performance. However, the conditional knockout of Drp1 lead to dilated heart disease, whereas the conditional knockout of Mfn1/Mfn2 lead to cardiac hypertrophy [116, 118]. Drp-1, together with Fis-1, functions to ensure equal division of the number of mitochondria during cell division and mediate the clearance of damaged mitochondria by mitochondrial autophagy. Mitochondrial fusion, on the other hand, functions in mitochondrial repair and regeneration. When mitochondrial fission and fusion genes are intact, damaged mitochondria can be appropriately cleared to maintain the mitochondrial renewal cycle. However, when fission gene mediated triage is interrupted, fusion transitions from regeneration to contamination [119].

*PTEN* (a tumor suppressor gene)-induced putative kinase 1 (PINK1)/Parkin pathway has been shown to play an important role in removing damaged mitochondria. One study indicated that myocardial mitophagy is significantly reduced in diabetic rats [119], activation of PINK1/Parkin mediated mitophagy improved myocardial mitochondrial function and block ventricular remodeling caused by diabetes [120]. Significant left ventricular dysfunction and signs of pathological cardiac hypertrophy were observed at 2 months of age in *PINK1* knockout mice [121]. *Parkin* knockout mice exhibit excessive cardiac hypertrophy in response to TAC surgery [122, 123].

**Fig. 2.**
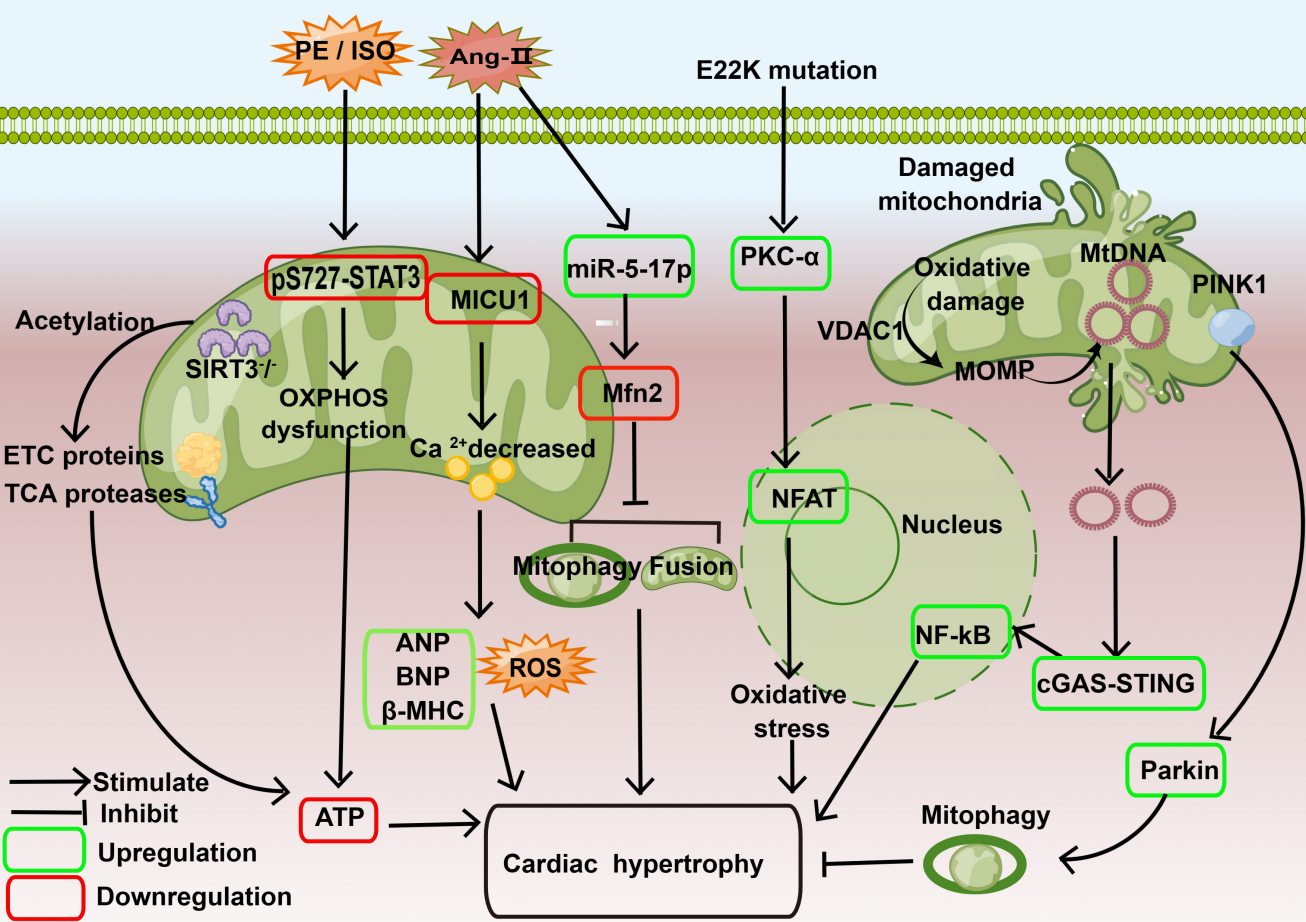
**Mechanisms by which mitochondria are involved in regulating cardiac hypertrophy through energy control, oxidative stress, mitochondrial DNA, mitochondrial dynamics, and more**. In the *SIRT3* knockout mouse model, three proteases involved in the TCA cycle and 50 proteins involved in the formation of ETC subunits are impaired, and the energy supply to cardiomyocytes is reduced, leading to the development of cardiac hypertrophy. In H9C2 cell hypertrophy model induced by PE and isoproterenol ISO, the phosphorylation level of p-S727-STAT3 and the expression of STAT3 were decreased, resulting in impaired interaction between p-S727-STAT3 and mitochondrial complexes and decreased expression of ETC complexes II and III. The OXPHOS system fails to generate sufficient amounts of ATP and induces cardiac hypertrophy. In the *in vitro* cardiomyocyte hypertrophy model of neonatal mice induced by Ang-II, the expression of MICU1 was decreased, and the mitochondrial membrane potential was abnormal, ROS production was increased, and the protein expression of ANP, BNP and β-MHC was increased, leading to cardiac hypertrophy. In the Ang-II-induced rat model, the expression of *miR-5-17p* is up-regulated and the expression of Mfn2 protein is down-regulated in myocardial tissue, which leads to a reduction of mitochondrial fusion and autophagy and causes cardiac hypertrophy. In a mouse model of ventricular hypertrophy caused by *E22K *mutation, the expression of PKC/NFAT (nuclear factor of activated T cells) axis is increased and oxidative stress is enhanced, leading to cardiac hypertrophy. In the hearts of CKD mice, mitochondrial oxidative damage increases MOMP through VDAC1, leading to the release of mtDNA into the cytoplasm and activation of STING-NF-κB signaling pathway, resulting in CKD-induced cardiac hypertrophy. Activation of the PINK1/Parkin pathway enhances mitophagy, improves myocardial mitochondrial function, blocks diabetic-induced ventricular remodeling, and improves ventricular hypertrophy. Ang-II, angiotensin II; ANP, atrial natriuretic peptide; ATP, adenosine triphosphate; BNP, brain natriuretic peptide; β-MHC, beta-myosin heavy chain; CKD, chronic kidney disease; ETC, electron transport chain; ISO, isoproterenol; Mfn2, mitofusin 2 protein; MICUI, mitochondrial Calcium Uptake protein 1; MOMP, mitochondrial outer membrane permeabilization; *MtDNA*, mitochondrial *DNA*; NFAT, nuclear factor of activated T cells; OXPHOS, oxidative phosphorylationl; PE, phenylephrine; PINK1/Parkin, phosphatase and tensin homolog deleted. on chromosome ten-induced putative kinase 1/Parkin; PKC, protein kinase C; pS727-STAT3, serine-phosphorylated STAT3; ROS, reactive oxygen species; STAT3, signal transducer and activator of transcription 3; STING, stimulator of interferon genes; TCA, tricarboxylic acid cycle; VDAC1, voltage-dependent anion channel 1; E22K, glutamic acid to lysine substitution at position 22; NF-κB, nuclear factor kappa B; cGAS, Cyclic guanosine monophosphate-adenosine monophosphate synthase; SIRT3, sirtuin 3.

The mechanism by which mitochondria are thought to participate in the regulation of cardiac hypertrophy is shown in Fig. 2.

## 4. Treatment Strategies 

Continuous research has been conducted to seek treatment strategies for heart disease based on mitochondrial diseases. Maneechote *et al*. [124] reported on the administration of mitochondrial fusion promoter (M1) alone or in combination with a Drp1 inhibitor (Mdivi-1), which significantly attenuated cardiac mitochondrial ROS production, membrane depolarization, swelling, and dynamic imbalance, and improved arrhythmias in prediabetic rats induced by ischemia-reperfusion.

Graham *et al*. [125] used oral administration of the mitochondrial-targeted antioxidant Q10 in stroke-prone spontaneously hypertensive rats (SHRSP) to prevent the development of hypertension and reduce cardiac hypertrophy. Diazoxide can avoid mitochondrial damage by opening a mitochondrial ATP-sensitive $K^{+}$ channel, thereby improving isoproterenol induced cardiac hypertrophy in mice [126].

Studies have shown that transient receptor potential vanilla-like type 1 (TRPV1) can promote the formation of mitochondria-associated ER membranes and protect mitochondrial function through the AMP-activated protein kinase/mfn2 pathway in cardiomyocytes, thereby treating cardiac hypertrophy induced by phenyloadrenaline [127].

EUK-1340 is a superoxide dismutase and catalase mimetic, which can prevent cardiomyocyte hypertrophy and fibrosis by targeting mitochondria to remove ROS [128]. Rheumatoid palmatum L. modulates mitochondrial SIRT3 signaling in mouse models of aortic constriction or isoproterenol induced cardiac hypertrophy and in phenylephrine injured cardiomyocytes, thus protecting the mitochondria *in vivo* or *in vitro* and treating cardiac hypertrophy [129].

The therapeutic strategies and experimental models are shown in Table 1 (Ref. [124, 125, 127, 129]).

**Table 1.** **Mitochondria-targeted therapeutic strategies and experimental models**.

Animal model	Treatment	Outcome	Reference(s)
Ischemia-reperfusion injury in prediabetic rats	Mitochondrial fusion promoter (M1) alone or in combination with Drp1 inhibitor (Mdivi-1) was administered before ischemia, during ischemia, or at the onset of reperfusion	Attenuated mitochondrial ROS production, membrane depolarization, swelling, and dynamic imbalance in the heart, resulting in arrhythmias and reduced infarct size, thereby improving left ventricular function in prediabetic rats	[124]
SHRSP	Oral administration of the mitochondria-targeting antioxidant mitochondrial Q10 prevented the development of hypertension in SHRSP	Reduced cardiac hypertrophy in rats	[125]
*In vitro* model of cardiac hypertrophy in TRPV1-treated phenylephrine treated mice	TRPV1 can promote the formation of MAM and protect mitochondrial function through AMPK/MFN2 pathway in cardiomyocytes	Treatment of cardiac hypertrophy	[127]
Mouse models of cardiac hypertrophy induced by aortic coarctation or isoproterenol	Palmar rhubarb regulates mitochondrial SIRT3 signaling in cardiac hypertrophyrats and mitochondrial SIRT3 signaling in phenylephrine injured cardiomyocytes	Delayed cardiac hypertrophy in mice	[129]

TRPV1, transient receptor potential vanilla-like type 1; MAM, mitochondria membranes; SIRT3, Sirtuin 3; ROS, reactive oxygen species; SHRSP, the stroke-prone spontaneously hypertensive rat.

## 5. Conclusions and Perspectives

To better understand the specific effects of mitochondrial dysfunction on heart disease, we elaborated the mechanisms of mitochondrial dysfunction on the occurrence and development of arrhythmia and cardiac hypertrophy from mitochondrial energy disorders, oxidative stress, and *mtDNA* abnormalities, mitochondrial autophagy and mitochondrial dynamics. In addition, we discussed the treatment strategies based on the relevant mechanisms we have summarized in relevant aspects. About ninety percent of the energy required for normal functioning of the heart is provided by the mitochondria of cardiomyocytes. ATP generated through OXPHOS to meet the energy demand of the heart [77, 78]. Abnormal generation of cardiac mitochondrial energy may lead to arrhythmia and cardiac hypertrophy ATP depletion may increase the possibility of the occurrence of arrhythmia [29]. The dysfunction of ETC complex subunits, the decreased activity of enzymes related to mitochondrial energy metabolism, as well as the reduction of OXPHOS related proteins, have been shown to significantly affect ATP production and be associated with the occurrence and development of arrhythmia [30, 31, 34]. In hypertrophic cardiac disease, protein acetylation related to mitochondrial energy metabolism increases and mitochondrial complex expression decreases; this leads to abnormal ATP production and the occurrence and development of cardiac hypertrophy [87, 89]. Yoo *et al*. [38] found that the atrial tissues of dogs receiving RAP showed increased mitochondrial ROS content and NADPH oxidase 2 activity. In animal models of arrhythmia, RyR2 overactivity and SR ${\mathrm{Ca}}^{2+}$ leakage lead to increased mitochondrial ROS emissions, supporting the relationship between mitochondria and ER/SR and their involvement in the occurrence and development of arrhythmia [42, 43]. Studies have shown that activated PKC and NFAT are related to the sensitization of mitochondrial stress signals. Stress factors, such as the depletion of active oxygen, drugs, or *mtDNA*, lead to a reduction in mitochondrial ${\mathrm{Ca}}^{2+}$ uptake and cytoplasmic ${\mathrm{Ca}}^{2+}$ overload, activate cellular PKC and nuclear response factors NFAT and cAMP response element-binding protein, and promote ventricular hypertrophy [93]. Knocking out Trx1, a protein that maintains redox stability in the body, can lead to increased cellular oxidative stress, as well as the appearance of cardiomyocyte hypertrophy [95]. MlCU1 is down-regulated in db/db mouse hearts, which is associated with development of cardiac hypertrophy and myocardial apoptosis. Reconstitution of MICU1 significantly reduced myocardial fibrosis, thereby inhibiting the progression of diabetic cardiomyopathy [98]. In a rat model of pneumonia-related sepsis, mitochondrial-targeted vitamin E therapy effectively eliminated mitochondrial ROS, protected mitochondria from lipid and protein peroxidation, reduced tissue inflammation, and improved cardiac function during sepsis [102]. Antioxidants have also shown to improve mitochondrial oxygen consumption and ATP production and prevent endotoxin-induced mitochondrial abnormalities [130]. Hamilton *et al*. [23] summarized several possible strategies against mitochondrial ${\mathrm{Ca}}^{2+}$ as a way to reduce cytoplasmic ${\mathrm{Ca}}^{2+}$ and improve arrhythmia disease . Increasing the uptake of mitochondrial ${\mathrm{Ca}}^{2+}$ from the cytoplasm [131] and inhibiting mitochondrial ${\mathrm{Ca}}^{2+}$ outflow [132, 133, 134] pose the risk of increasing mitochondrial ROS production [87, 88]; superior benefits can be gained by inhibiting the uptake of mitochondrial ${\mathrm{Ca}}^{2+}$ using MICU inhibitors (R360 [40] and Ru265 [135]) or small-conductance ${\mathrm{Ca}}^{2+}$-activated $K^{+}$ channel enhancers [42, 136]. In contrast, ${\mathrm{Ca}}^{2+}$channel antagonists targeting ${\mathrm{Ca}}^{2+}$ treatment of cardiomyocytes have been shown to be effective in the management of arrhythmias and related heart diseases [137, 138]. Cardiac hypertrophy remains an important cause of sudden death in young individuals. Current treatments reduce myocardial cell uptake of ${\mathrm{Ca}}^{2+}$; thus, relieving cardiac symptoms. ${\mathrm{Ca}}^{2+}$desensitizers have been used to prevent cardiac hypertrophy in mouse models and [101] may be effective in treating cardiac hypertrophy [139]. Studies have shown that a decrease in the copy number of *mtDNA*, mutations in *mtDNA* fragments, and *mtDNA* released into the cytoplasm after oxidative stress can cause both arrhythmia and cardiac hypertrophy [44, 101, 105, 108]. In various heart diseases, cardiac dysfunction caused by mitochondrial homeostasis imbalance is very common. Off balance in mitochondrial dynamics or mitochondrial autophagy may lead to the occurrence and progression of arrhythmia and ventricular hypertrophy diseases. Maintaining mitochondrial homeostasis in the heart may one of important strategy for treatment for heart diseases. In research on the involvement of mitochondria in the regulation of heart disease, it is particularly important to screen biomarkers related to mitochondrial damage, which will provide guidance for the diagnosis and treatment of mitochondrial related heart disease.

## Abbreviations

AA, Ascorbic acid; AF, Atrial fibrillation; ATP, Adenosine triphosphate; ${\mathrm{Ca}}^{2+}$, Calcium ion; CKD, Chronic kidney disease; CPVT, Catecholaminergic polymorphic ventricular tachycardia; Cyt-c, Cytochrome-c; DRP1, Dynamin-related protein 1; ER, Endoplasmic reticulum; ETC, Electron transport chain; HCM, Hypertrophic cardiomyopathy; HF, Heart failure; IMS, Intermembrane mitochondrial space; Mfn1, Mitofusin-1; Mfn2, Mitofusin-2; MHC, Myosin heavy chain; MICU1, Mitochondrial calcium uptake 1; *MtDNA*, Mitochondrial *DNA*; mutUNG1, mutant uracil-DNA glycosylase 1; NFAT, Nuclear factor of activated T cell; OPA1, Optic atrophy protein 1; OXPHOS, Oxidative phosphorylation; PINK1, *PTEN*-induced putative kinase 1; PKC, Protein kinase C; PTM, Post-translational modifications; RAP, Rapid atrial pacing; ROS, Reactive oxygen species; RyR2, Ryanodine receptor 2; SHRSP, Spontaneously hypertensive rats; SIRT3, Sirtuin 3; SR, Sarcoplasmic reticulum; STAT3, Signal transducer and activator of transcription 3; STING, Stimulator of interferon genes; TAC, Transverse aortic constriction; *TECRL*, Trans-2,3-enoyl-CoA reductase-like; TRPV1, Transient receptor potential vanilla-like type 1; Trx, Thioredoxin; VMs, Ventricular myocytes; cGAS, Cyclic guanosine monophosphate-adenosine monophosphate synthase; E22K, glutamic acid to lysine substitution at position 22; EMD,Emerin;FIS1, mitochondrial fission protein 1; IKH, acetylcholine-dependent K current; KO, knockout; M1, mitochondrial fusion promoter; MDA, Malondialdehyde; Mdivi-1, Drp1 inhibitor; MFF, mitochondrial fission factor; NF-κB, Nuclear factor kappa B; NLRP3, nucleotide-binding oligomerization domain-like receptor pyrin domain containing 3; PM2.5, particulate matter 2.5; VBIT-4, voltage dependent anion channel 1-mediated mitochondrial outer membrane permeability inhibitor.

## References

[1] Sygitowicz G, Sitkiewicz D (2022). Mitochondrial quality control: the role in cardiac injury. *Frontiers in Bioscience-Landmark*.

[2] El-Hattab AW, Craigen WJ, Scaglia F (2017). Mitochondrial DNA maintenance defects. *Biochimica et Biophysica Acta. Molecular Basis of Disease*.

[3] Shadel GS, Horvath TL (2015). Mitochondrial ROS signaling in organismal homeostasis. *Cell*.

[4] Maulik SK, Kumar S (2012). Oxidative stress and cardiac hypertrophy: a review. *Toxicology Mechanisms and Methods*.

[5] Tsutsui H, Kinugawa S, Matsushima S (2011). Oxidative stress and heart failure. *American Journal of Physiology. Heart and Circulatory Physiology*.

[6] Protasoni M, Zeviani M (2021). Mitochondrial Structure and Bioenergetics in Normal and Disease Conditions. *International Journal of Molecular Sciences*.

[7] Mammucari C, Raffaello A, Vecellio Reane D, Gherardi G, De Mario A, Rizzuto R (2018). Mitochondrial calcium uptake in organ physiology: from molecular mechanism to animal models. *Pflugers Archiv: European Journal of Physiology*.

[8] Rossi A, Pizzo P, Filadi R (2019). Calcium, mitochondria and cell metabolism: A functional triangle in bioenergetics. *Biochimica et Biophysica Acta. Molecular Cell Research*.

[9] Bock FJ, Tait SWG (2020). Mitochondria as multifaceted regulators of cell death. *Nature Reviews. Molecular Cell Biology*.

[10] Taanman JW (1999). The mitochondrial genome: structure, transcription, translation and replication. *Biochimica et Biophysica Acta*.

[11] Yan C, Duanmu X, Zeng L, Liu B, Song Z (2019). Mitochondrial DNA: Distribution, Mutations, and Elimination. *Cells*.

[12] Vona R, Mileo AM, Matarrese P (2021). Microtubule-Based Mitochondrial Dynamics as a Valuable Therapeutic Target in Cancer. *Cancers*.

[13] Disatnik MH, Ferreira JCB, Campos JC, Gomes KS, Dourado PMM, Qi X (2013). Acute inhibition of excessive mitochondrial fission after myocardial infarction prevents long-term cardiac dysfunction. *Journal of the American Heart Association*.

[14] Figueira TR, Barros MH, Camargo AA, Castilho RF, Ferreira JCB, Kowaltowski AJ (2013). Mitochondria as a source of reactive oxygen and nitrogen species: from molecular mechanisms to human health. *Antioxidants & Redox Signaling*.

[15] Bayeva M, Gheorghiade M, Ardehali H (2013). Mitochondria as a therapeutic target in heart failure. *Journal of the American College of Cardiology*.

[16] Kasahara A, Cipolat S, Chen Y, Dorn GW, Scorrano L (2013). Mitochondrial fusion directs cardiomyocyte differentiation via calcineurin and Notch signaling. *Science (New York, N.Y.)*.

[17] O’Rourke B, Cortassa S, Aon MA (2005). Mitochondrial ion channels: gatekeepers of life and death. *Physiology (Bethesda, Md.)*.

[18] Hasan P, Saotome M, Ikoma T, Iguchi K, Kawasaki H, Iwashita T (2018). Mitochondrial fission protein, dynamin-related protein 1, contributes to the promotion of hypertensive cardiac hypertrophy and fibrosis in Dahl-salt sensitive rats. *Journal of Molecular and Cellular Cardiology*.

[19] Liu X, Guo C, Zhang Q (2023). Novel insights into the involvement of mitochondrial fission/fusion in heart failure: From molecular mechanisms to targeted therapies. *Cell Stress & Chaperones*.

[20] Sazonova MA, Sinyov VV, Barinova VA, Ryzhkova AI, Zhelankin AV, Postnov AY (2015). Mosaicism of mitochondrial genetic variation in atherosclerotic lesions of the human aorta. *BioMed Research International*.

[21] Maack C, O’Rourke B (2007). Excitation-contraction coupling and mitochondrial energetics. *Basic Research in Cardiology*.

[22] Nikolaienko R, Bovo E, Zima AV (2018). Redox Dependent Modifications of Ryanodine Receptor: Basic Mechanisms and Implications in Heart Diseases. *Frontiers in Physiology*.

[23] Hamilton S, Terentyeva R, Clements RT, Belevych AE, Terentyev D (2021). Sarcoplasmic reticulum-mitochondria communication; implications for cardiac arrhythmia. *Journal of Molecular and Cellular Cardiology*.

[24] Eisner DA, Caldwell JL, Kistamás K, Trafford AW (2017). Calcium and Excitation-Contraction Coupling in the Heart. *Circulation Research*.

[25] Huizar JF, Ellenbogen KA, Tan AY, Kaszala K (2019). Arrhythmia-Induced Cardiomyopathy: JACC State-of-the-Art Review. *Journal of the American College of Cardiology*.

[26] Lippi G, Sanchis-Gomar F, Cervellin G (2021). Global epidemiology of atrial fibrillation: An increasing epidemic and public health challenge. *International Journal of Stroke*.

[27] Holmberg M, Holmberg S, Herlitz J (1999). The problem of out-of-hospital cardiac-arrest prevalence of sudden death in Europe today. *The American Journal of Cardiology*.

[28] Yang KC, Bonini MG, Dudley SC (2014). Mitochondria and arrhythmias. *Free Radical Biology & Medicine*.

[29] Samuel TJ, Lai S, Schär M, Wu KC, Steinberg AM, Wei AC (2022). Myocardial ATP depletion detected noninvasively predicts sudden cardiac death risk in patients with heart failure. *JCI Insight*.

[30] Emelyanova L, Ashary Z, Cosic M, Negmadjanov U, Ross G, Rizvi F (2016). Selective downregulation of mitochondrial electron transport chain activity and increased oxidative stress in human atrial fibrillation. *American Journal of Physiology. Heart and Circulatory Physiology*.

[31] Tu T, Zhou S, Liu Z, Li X, Liu Q (2014). Quantitative proteomics of changes in energy metabolism-related proteins in atrial tissue from valvular disease patients with permanent atrial fibrillation. *Circulation Journal*.

[32] Kanaan GN, Patten DA, Redpath CJ, Harper ME (2019). Atrial Fibrillation Is Associated With Impaired Atrial Mitochondrial Energetics and Supercomplex Formation in Adults With Type 2 Diabetes. *Canadian Journal of Diabetes*.

[33] Montaigne D, Marechal X, Lefebvre P, Modine T, Fayad G, Dehondt H (2013). Mitochondrial dysfunction as an arrhythmogenic substrate: a translational proof-of-concept study in patients with metabolic syndrome in whom post-operative atrial fibrillation develops. *Journal of the American College of Cardiology*.

[34] Hou C, Jiang X, Zhang H, Zheng J, Qiu Q, Zhang Y (2022). TECRL deficiency results in aberrant mitochondrial function in cardiomyocytes. *Communications Biology*.

[35] Xie L, Hou C, Jiang X, Zhao J, Li Y, Xiao T (2019). A compound heterozygosity of Tecrl gene confirmed in a catecholaminergic polymorphic ventricular tachycardia family. *European Journal of Medical Genetics*.

[36] Caterine MR, Spencer KT, Pagan-Carlo LA, Smith RS, Buettner GR, Kerber RE (1996). Direct current shocks to the heart generate free radicals: an electron paramagnetic resonance study. *Journal of the American College of Cardiology*.

[37] Tsai MS, Huang CH, Tsai CY, Chen HW, Cheng HJ, Hsu CY (2014). Combination of intravenous ascorbic acid administration and hypothermia after resuscitation improves myocardial function and survival in a ventricular fibrillation cardiac arrest model in the rat. *Academic Emergency Medicine: Official Journal of the Society for Academic Emergency Medicine*.

[38] Yoo S, Pfenniger A, Hoffman J, Zhang W, Ng J, Burrell A (2020). Attenuation of Oxidative Injury With Targeted Expression of NADPH Oxidase 2 Short Hairpin RNA Prevents Onset and Maintenance of Electrical Remodeling in the Canine Atrium: A Novel Gene Therapy Approach to Atrial Fibrillation. *Circulation*.

[39] Sharma VK, Ramesh V, Franzini-Armstrong C, Sheu SS (2000). Transport of Ca2+ from sarcoplasmic reticulum to mitochondria in rat ventricular myocytes. *Journal of Bioenergetics and Biomembranes*.

[40] Liu T, O’Rourke B (2008). Enhancing mitochondrial Ca2+ uptake in myocytes from failing hearts restores energy supply and demand matching. *Circulation Research*.

[41] Ruiz-Meana M, Fernandez-Sanz C, Garcia-Dorado D (2010). The SR-mitochondria interaction: a new player in cardiac pathophysiology. *Cardiovascular Research*.

[42] Venetucci LA, Trafford AW, O’Neill SC, Eisner DA (2008). The sarcoplasmic reticulum and arrhythmogenic calcium release. *Cardiovascular Research*.

[43] Hamilton S, Terentyeva R, Martin B, Perger F, Li J, Stepanov A (2020). Increased RyR2 activity is exacerbated by calcium leak-induced mitochondrial ROS. *Basic Research in Cardiology*.

[44] Zhao D, Bartz TM, Sotoodehnia N, Post WS, Heckbert SR, Alonso A (2020). Mitochondrial DNA copy number and incident atrial fibrillation. *BMC Medicine*.

[45] Lin PH, Lee SH, Su CP, Wei YH (2003). Oxidative damage to mitochondrial DNA in atrial muscle of patients with atrial fibrillation. *Free Radical Biology & Medicine*.

[46] Park HW, Ahn Y, Jeong MH, Cho JG, Park JC, Kang JC (2007). Chronic atrial fibrillation associated with somatic mitochondrial DNA mutations in human atrial tissue. *Journal of Clinical Pathology*.

[47] Dzeshka MS, Lip GYH, Snezhitskiy V, Shantsila E (2015). Cardiac Fibrosis in Patients With Atrial Fibrillation: Mechanisms and Clinical Implications. *Journal of the American College of Cardiology*.

[48] Du Z, Zhu T, Lin M, Bao Y, Qiao J, Lv G (2022). A novel mutation in human EMD gene and mitochondrial dysfunction in emerin knockdown cardiomyocytes. *Journal of Cellular and Molecular Medicine*.

[49] Della Grazia PM, Klugmann S, Morgera T, Salvi A, Pandullo C, Camerini F (1986). Reperfusion ventricular arrhythmias during intracoronary thrombolysis. *European Heart Journal*.

[50] Lahnwong S, Palee S, Apaijai N, Sriwichaiin S, Kerdphoo S, Jaiwongkam T (2020). Acute dapagliflozin administration exerts cardioprotective effects in rats with cardiac ischemia/reperfusion injury. *Cardiovascular Diabetology*.

[51] Liu J, Wang P, Zou L, Qu J, Litovsky S, Umeda P (2014). High-fat, low-carbohydrate diet promotes arrhythmic death and increases myocardial ischemia-reperfusion injury in rats. *American Journal of Physiology. Heart and Circulatory Physiology*.

[52] Chen D, Li X, Zhang L, Zhu M, Gao L (2018). A high-fat diet impairs mitochondrial biogenesis, mitochondrial dynamics, and the respiratory chain complex in rat myocardial tissues. *Journal of Cellular Biochemistry*.

[53] Murphy KR, Baggett B, Cooper LL, Lu Y, O-Uchi J, Sedivy JM (2019). Enhancing Autophagy Diminishes Aberrant Ca^2+^ Homeostasis and Arrhythmogenesis in Aging Rabbit Hearts. *Frontiers in Physiology*.

[54] Abumayyaleh M, Demmer J, Krack C, Pilsinger C, El-Battrawy I, Aweimer A (2023). Incidence of atrial and ventricular arrhythmias in obese patients with heart failure with reduced ejection fraction treated with sacubitril/valsartan. *Diabetes, Obesity & Metabolism*.

[55] Chen Y, Liu Y, Dorn GW (2011). Mitochondrial fusion is essential for organelle function and cardiac homeostasis. *Circulation Research*.

[56] Ashrafian H, Docherty L, Leo V, Towlson C, Neilan M, Steeples V (2010). A mutation in the mitochondrial fission gene Dnm1l leads to cardiomyopathy. *PLoS Genetics*.

[57] Ikeda Y, Shirakabe A, Maejima Y, Zhai P, Sciarretta S, Toli J (2015). Endogenous Drp1 mediates mitochondrial autophagy and protects the heart against energy stress. *Circulation Research*.

[58] Cassidy-Stone A, Chipuk JE, Ingerman E, Song C, Yoo C, Kuwana T (2008). Chemical inhibition of the mitochondrial division dynamin reveals its role in Bax/Bak-dependent mitochondrial outer membrane permeabilization. *Developmental Cell*.

[59] Ong SB, Subrayan S, Lim SY, Yellon DM, Davidson SM, Hausenloy DJ (2010). Inhibiting mitochondrial fission protects the heart against ischemia/reperfusion injury. *Circulation*.

[60] Ishaq L (2015). Role of Mitofusin-2 and Mitochondria-Associated Membrane in Arrhythmogenesis. https://ruor.uottawa.ca/bitstream/10393/32668/1/Ishaq%2C%20Laith_UROP%20Poster.pdf.

[61] Brook RD, Rajagopalan S, Pope CA, Brook JR, Bhatnagar A, Diez-Roux AV (2010). Particulate matter air pollution and cardiovascular disease: An update to the scientific statement from the American Heart Association. *Circulation*.

[62] Li R, Kou X, Geng H, Xie J, Tian J, Cai Z (2015). Mitochondrial damage: an important mechanism of ambient PM2.5 exposure-induced acute heart injury in rats. *Journal of Hazardous Materials*.

[63] Sivakumar B, Kurian GA (2022). Inhalation of PM_2.5_ from diesel exhaust promote impairment of mitochondrial bioenergetics and dysregulate mitochondrial quality in rat heart: implications in isoproterenol-induced myocardial infarction model. *Inhalation Toxicology*.

[64] Shao Q, Meng L, Lee S, Tse G, Gong M, Zhang Z (2019). Empagliflozin, a sodium glucose co-transporter-2 inhibitor, alleviates atrial remodeling and improves mitochondrial function in high-fat diet/streptozotocin-induced diabetic rats. *Cardiovascular Diabetology*.

[65] Wang S, Zhao Z, Feng X, Cheng Z, Xiong Z, Wang T (2018). Melatonin activates Parkin translocation and rescues the impaired mitophagy activity of diabetic cardiomyopathy through Mst1 inhibition. *Journal of Cellular and Molecular Medicine*.

[66] Sommese L, Valverde CA, Blanco P, Castro MC, Rueda OV, Kaetzel M (2016). Ryanodine receptor phosphorylation by CaMKII promotes spontaneous Ca(2+) release events in a rodent model of early stage diabetes: The arrhythmogenic substrate. *International Journal of Cardiology*.

[67] Liu M, Liu H, Dudley SC (2010). Reactive oxygen species originating from mitochondria regulate the cardiac sodium channel. *Circulation Research*.

[68] Yang R, Ernst P, Song J, Liu XM, Huke S, Wang S (2018). Mitochondrial-Mediated Oxidative Ca^2+^/Calmodulin-Dependent Kinase II Activation Induces Early Afterdepolarizations in Guinea Pig Cardiomyocytes: An *In Silico* Study. *Journal of the American Heart Association*.

[69] LaRocca TJ, Fabris F, Chen J, Benhayon D, Zhang S, McCollum L (2012). Na+/Ca2+ exchanger-1 protects against systolic failure in the Akitains2 model of diabetic cardiomyopathy via a CXCR4/NF-κB pathway. *American Journal of Physiology. Heart and Circulatory Physiology*.

[70] Mesubi OO, Anderson ME (2016). Atrial remodelling in atrial fibrillation: CaMKII as a nodal proarrhythmic signal. *Cardiovascular Research*.

[71] Samak M, Fatullayev J, Sabashnikov A, Zeriouh M, Schmack B, Farag M (2016). Cardiac Hypertrophy: An Introduction to Molecular and Cellular Basis. *Medical Science Monitor Basic Research*.

[72] Gallo S, Vitacolonna A, Bonzano A, Comoglio P, Crepaldi T (2019). ERK: A Key Player in the Pathophysiology of Cardiac Hypertrophy. *International Journal of Molecular Sciences*.

[73] Rosca MG, Tandler B, Hoppel CL (2013). Mitochondria in cardiac hypertrophy and heart failure. *Journal of Molecular and Cellular Cardiology*.

[74] Borer JS (2004). Left ventricular hypertrophy in hypertrophic cardiomyopathy: what’s in a phenotype. *Journal of the American College of Cardiology*.

[75] Alharbi HO, Hardyman MA, Cull JJ, Markou T, Cooper STE, Glennon PE (2022). Cardiomyocyte BRAF is a key signalling intermediate in cardiac hypertrophy in mice. *Clinical Science (London, England: 1979)*.

[76] Mitra A, Basak T, Datta K, Naskar S, Sengupta S, Sarkar S (2013). Role of α-crystallin B as a regulatory switch in modulating cardiomyocyte apoptosis by mitochondria or endoplasmic reticulum during cardiac hypertrophy and myocardial infarction. *Cell Death & Disease*.

[77] Mootha VK, Arai AE, Balaban RS (1997). Maximum oxidative phosphorylation capacity of the mammalian heart. *The American Journal of Physiology*.

[78] Lemieux H, Hoppel CL (2009). Mitochondria in the human heart. *Journal of Bioenergetics and Biomembranes*.

[79] Tahrir FG, Langford D, Amini S, Mohseni Ahooyi T, Khalili K (2019). Mitochondrial quality control in cardiac cells: Mechanisms and role in cardiac cell injury and disease. *Journal of Cellular Physiology*.

[80] Stram AR, Payne RM (2016). Post-translational modifications in mitochondria: protein signaling in the powerhouse. *Cellular and Molecular Life Sciences: CMLS*.

[81] Comità S, Femmino S, Thairi C, Alloatti G, Boengler K, Pagliaro P (2021). Regulation of STAT3 and its role in cardioprotection by conditioning: focus on non-genomic roles targeting mitochondrial function. *Basic Research in Cardiology*.

[82] Sheeran FL, Pepe S (2017). Mitochondrial Bioenergetics and Dysfunction in Failing Heart. *Advances in Experimental Medicine and Biology*.

[83] Sheeran FL, Pepe S (2016). Posttranslational modifications and dysfunction of mitochondrial enzymes in human heart failure. *American Journal of Physiology. Endocrinology and Metabolism*.

[84] Schwer B, North BJ, Frye RA, Ott M, Verdin E (2002). The human silent information regulator (Sir)2 homologue hSIRT3 is a mitochondrial nicotinamide adenine dinucleotide-dependent deacetylase. *The Journal of Cell Biology*.

[85] Lombard DB, Alt FW, Cheng HL, Bunkenborg J, Streeper RS, Mostoslavsky R (2007). Mammalian Sir2 homolog SIRT3 regulates global mitochondrial lysine acetylation. *Molecular and Cellular Biology*.

[86] Boardman NT, Migally B, Pileggi C, Parmar GS, Xuan JY, Menzies K (2021). Glutaredoxin-2 and Sirtuin-3 deficiencies impair cardiac mitochondrial energetics but their effects are not additive. *Biochimica et Biophysica Acta. Molecular Basis of Disease*.

[87] Koentges C, Pfeil K, Schnick T, Wiese S, Dahlbock R, Cimolai MC (2015). SIRT3 deficiency impairs mitochondrial and contractile function in the heart. *Basic Research in Cardiology*.

[88] Kunisada K, Tone E, Fujio Y, Matsui H, Yamauchi-Takihara K, Kishimoto T (1998). Activation of gp130 transduces hypertrophic signals via STAT3 in cardiac myocytes. *Circulation*.

[89] Jeong K, Kwon H, Min C, Pak Y (2009). Modulation of the caveolin-3 localization to caveolae and STAT3 to mitochondria by catecholamine-induced cardiac hypertrophy in H9c2 cardiomyoblasts. *Experimental & Molecular Medicine*.

[90] Bugger H, Schwarzer M, Chen D, Schrepper A, Amorim PA, Schoepe M (2010). Proteomic remodelling of mitochondrial oxidative pathways in pressure overload-induced heart failure. *Cardiovascular Research*.

[91] Ni Y, Deng J, Liu X, Li Q, Zhang J, Bai H (2021). Echinacoside reverses myocardial remodeling and improves heart function via regulating SIRT1/FOXO3a/MnSOD axis in HF rats induced by isoproterenol. *Journal of Cellular and Molecular Medicine*.

[92] Szczesna D, Ghosh D, Li Q, Gomes AV, Guzman G, Arana C (2001). Familial hypertrophic cardiomyopathy mutations in the regulatory light chains of myosin affect their structure, Ca2+ binding, and phosphorylation. *The Journal of Biological Chemistry*.

[93] Wang H, Lin Y, Zhang R, Chen Y, Ji W, Li S (2022). Programmed Exercise Attenuates Familial Hypertrophic Cardiomyopathy in Transgenic E22K Mice *via* Inhibition of PKC-α/NFAT Pathway. *Frontiers in Cardiovascular Medicine*.

[94] Wang J, Zhou J, Wang C, Fukunaga A, Li S, Yodoi J (2022). Thioredoxin-1: A Promising Target for the Treatment of Allergic Diseases. *Frontiers in Immunology*.

[95] Andreadou I, Efentakis P, Frenis K, Daiber A, Schulz R (2021). Thiol-based redox-active proteins as cardioprotective therapeutic agents in cardiovascular diseases. *Basic Research in Cardiology*.

[96] Ott M, Robertson JD, Gogvadze V, Zhivotovsky B, Orrenius S (2002). Cytochrome c release from mitochondria proceeds by a two-step process. *Proceedings of the National Academy of Sciences of the United States of America*.

[97] Mallilankaraman K, Doonan P, Cárdenas C, Chandramoorthy HC, Müller M, Miller R (2012). MICU1 is an essential gatekeeper for MCU-mediated mitochondrial Ca(2+) uptake that regulates cell survival. *Cell*.

[98] Ji L, Liu F, Jing Z, Huang Q, Zhao Y, Cao H (2017). MICU1 Alleviates Diabetic Cardiomyopathy Through Mitochondrial Ca^2+^-Dependent Antioxidant Response. *Diabetes*.

[99] Yang Y, Du J, Xu R, Shen Y, Yang D, Li D (2020). Melatonin alleviates angiotensin-II-induced cardiac hypertrophy via activating MICU1 pathway. *Aging*.

[100] Dai DF, Johnson SC, Villarin JJ, Chin MT, Nieves-Cintrón M, Chen T (2011). Mitochondrial oxidative stress mediates angiotensin II-induced cardiac hypertrophy and Galphaq overexpression-induced heart failure. *Circulation Research*.

[101] Dia M, Gomez L, Thibault H, Tessier N, Leon C, Chouabe C (2020). Reduced reticulum-mitochondria Ca^2+^ transfer is an early and reversible trigger of mitochondrial dysfunctions in diabetic cardiomyopathy. *Basic Research in Cardiology*.

[102] Zhu R, Meng ZL, Chen L, Chen W, Wang H, Hong YQ (2016). Complete mitochondrial genome sequence and mutations of the cardiac hypertrophy model inbred rat strain (Muridae; Rattus). *Mitochondrial DNA. Part A, DNA Mapping, Sequencing, and Analysis*.

[103] Ide T, Tsutsui H, Hayashidani S, Kang D, Suematsu N, Nakamura K (2001). Mitochondrial DNA damage and dysfunction associated with oxidative stress in failing hearts after myocardial infarction. *Circulation Research*.

[104] Han W, Du C, Zhu Y, Ran L, Wang Y, Xiong J (2022). Targeting Myocardial Mitochondria-STING-Polyamine Axis Prevents Cardiac Hypertrophy in Chronic Kidney Disease. *JACC. Basic to Translational Science*.

[105] Ishikawa H, Barber GN (2008). STING is an endoplasmic reticulum adaptor that facilitates innate immune signalling. *Nature*.

[106] Hopfner KP, Hornung V (2020). Molecular mechanisms and cellular functions of cGAS-STING signalling. *Nature Reviews. Molecular Cell Biology*.

[107] Yan M, Li Y, Luo Q, Zeng W, Shao X, Li L (2022). Mitochondrial damage and activation of the cytosolic DNA sensor cGAS-STING pathway lead to cardiac pyroptosis and hypertrophy in diabetic cardiomyopathy mice. *Cell Death Discovery*.

[108] Kavli B, Slupphaug G, Mol CD, Arvai AS, Peterson SB, Tainer JA (1996). Excision of cytosine and thymine from DNA by mutants of human uracil-DNA glycosylase. *The EMBO Journal*.

[109] Valencik ML, McDonald JA (2001). Codon optimization markedly improves doxycycline regulated gene expression in the mouse heart. *Transgenic Research*.

[110] Lauritzen KH, Kleppa L, Aronsen JM, Eide L, Carlsen H, Haugen ØP (2015). Impaired dynamics and function of mitochondria caused by mtDNA toxicity leads to heart failure. *American Journal of Physiology. Heart and Circulatory Physiology*.

[111] Lamperth L, Dalakas MC, Dagani F, Anderson J, Ferrari R (1991). Abnormal skeletal and cardiac muscle mitochondria induced by zidovudine (AZT) in human muscle *in vitro* and in an animal model. *Laboratory Investigation; a Journal of Technical Methods and Pathology*.

[112] Sabbah HN, Gupta RC, Singh-Gupta V, Zhang K, Lanfear DE (2018). Abnormalities of Mitochondrial Dynamics in the Failing Heart: Normalization Following Long-Term Therapy with Elamipretide. *Cardiovascular Drugs and Therapy*.

[113] Sun D, Li C, Liu J, Wang Z, Liu Y, Luo C (2019). Expression Profile of microRNAs in Hypertrophic Cardiomyopathy and Effects of microRNA-20 in Inducing Cardiomyocyte Hypertrophy Through Regulating Gene *MFN2*. *DNA and Cell Biology*.

[114] Xu X, Su YL, Shi JY, Lu Q, Chen C (2021). MicroRNA-17-5p Promotes Cardiac Hypertrophy by Targeting Mfn2 to Inhibit Autophagy. *Cardiovascular Toxicology*.

[115] Fang L, Moore XL, Gao XM, Dart AM, Lim YL, Du XJ (2007). Down-regulation of mitofusin-2 expression in cardiac hypertrophy *in vitro* and *in vivo*. *Life Sciences*.

[116] Song M, Mihara K, Chen Y, Scorrano L, Dorn GW (2015). Mitochondrial fission and fusion factors reciprocally orchestrate mitophagic culling in mouse hearts and cultured fibroblasts. *Cell Metabolism*.

[117] Chang YW, Chang YT, Wang Q, Lin JJC, Chen YJ, Chen CC (2013). Quantitative phosphoproteomic study of pressure-overloaded mouse heart reveals dynamin-related protein 1 as a modulator of cardiac hypertrophy. *Molecular & Cellular Proteomics: MCP*.

[118] Wang S, Tan J, Miao Y, Zhang Q (2022). Mitochondrial Dynamics, Mitophagy, and Mitochondria-Endoplasmic Reticulum Contact Sites Crosstalk Under Hypoxia. *Frontiers in Cell and Developmental Biology*.

[119] Morales PE, Arias-Durán C, Ávalos-Guajardo Y, Aedo G, Verdejo HE, Parra V (2020). Emerging role of mitophagy in cardiovascular physiology and pathology. *Molecular Aspects of Medicine*.

[120] Tong M, Saito T, Zhai P, Oka SI, Mizushima W, Nakamura M (2019). Mitophagy Is Essential for Maintaining Cardiac Function During High Fat Diet-Induced Diabetic Cardiomyopathy. *Circulation Research*.

[121] Billia F, Hauck L, Konecny F, Rao V, Shen J, Mak TW (2011). PTEN-inducible kinase 1 (PINK1)/Park6 is indispensable for normal heart function. *Proceedings of the National Academy of Sciences of the United States of America*.

[122] Shires SE, Kubli DA, Gonzalez ER, Purcell NH, Gustafsson ÅB (2015). Parkin Contributes to the Development of Cardiac Hypertrophy in Response to Cardiac Pressure Overload. *Circulation Research*.

[123] Han K, Hassanzadeh S, Singh K, Menazza S, Nguyen TT, Stevens MV (2017). Parkin regulation of CHOP modulates susceptibility to cardiac endoplasmic reticulum stress. *Scientific Reports*.

[124] Maneechote C, Palee S, Kerdphoo S, Jaiwongkam T, Chattipakorn SC, Chattipakorn N (2022). Modulating mitochondrial dynamics attenuates cardiac ischemia-reperfusion injury in prediabetic rats. *Acta Pharmacologica Sinica*.

[125] Graham D, Huynh NN, Hamilton CA, Beattie E, Smith RAJ, Cochemé HM (2009). Mitochondria-targeted antioxidant MitoQ10 improves endothelial function and attenuates cardiac hypertrophy. *Hypertension (Dallas, Tex.: 1979)*.

[126] Lucas AM, Caldas FR, da Silva AP, Ventura MM, Leite IM, Filgueiras AB (2017). Diazoxide prevents reactive oxygen species and mitochondrial damage, leading to anti-hypertrophic effects. *Chemico-biological Interactions*.

[127] Wang Y, Li X, Xu X, Qu X, Yang Y (2022). Transient Receptor Potential Vanilloid Type 1 Protects Against Pressure Overload-Induced Cardiac Hypertrophy by Promoting Mitochondria-Associated Endoplasmic Reticulum Membranes. *Journal of Cardiovascular Pharmacology*.

[128] Redout EM, van der Toorn A, Zuidwijk MJ, van de Kolk CWA, van Echteld CJA, Musters RJP (2010). Antioxidant treatment attenuates pulmonary arterial hypertension-induced heart failure. *American Journal of Physiology. Heart and Circulatory Physiology*.

[129] Gao J, Zhang K, Wang Y, Guo R, Liu H, Jia C (2020). A machine learning-driven study indicates emodin improves cardiac hypertrophy by modulation of mitochondrial SIRT3 signaling. *Pharmacological Research*.

[130] Maneechote C, Palee S, Kerdphoo S, Jaiwongkam T, Chattipakorn SC, Chattipakorn N (2018). Differential temporal inhibition of mitochondrial fission by Mdivi-1 exerts effective cardioprotection in cardiac ischemia/reperfusion injury. *Clinical Science (London, England: 1979)*.

[131] Pathak T, Trebak M (2018). Mitochondrial Ca^2+^ signaling. *Pharmacology & Therapeutics*.

[132] Neumann JT, Diaz-Sylvester PL, Fleischer S, Copello JA (2011). CGP-37157 inhibits the sarcoplasmic reticulum Ca²+ ATPase and activates ryanodine receptor channels in striated muscle. *Molecular Pharmacology*.

[133] Liu T, Yang N, Sidor A, O’Rourke B (2021). MCU Overexpression Rescues Inotropy and Reverses Heart Failure by Reducing SR Ca^2+^ Leak. *Circulation Research*.

[134] García-Rivas GDJ, Carvajal K, Correa F, Zazueta C (2006). Ru360, a specific mitochondrial calcium uptake inhibitor, improves cardiac post-ischaemic functional recovery in rats *in vivo*. *British Journal of Pharmacology*.

[135] Nilius B, Hess P, Lansman JB, Tsien RW (1985). A novel type of cardiac calcium channel in ventricular cells. *Nature*.

[136] Curtis BM, Catterall WA (1984). Purification of the calcium antagonist receptor of the voltage-sensitive calcium channel from skeletal muscle transverse tubules. *Biochemistry*.

[137] Supinski GS, Murphy MP, Callahan LA (2009). MitoQ administration prevents endotoxin-induced cardiac dysfunction. *American Journal of Physiology. Regulatory, Integrative and Comparative Physiology*.

[138] Zang QS, Sadek H, Maass DL, Martinez B, Ma L, Kilgore JA (2012). Specific inhibition of mitochondrial oxidative stress suppresses inflammation and improves cardiac function in a rat pneumonia-related sepsis model. *American Journal of Physiology. Heart and Circulatory Physiology*.

[139] Schober T, Huke S, Venkataraman R, Gryshchenko O, Kryshtal D, Hwang HS (2012). Myofilament Ca sensitization increases cytosolic Ca binding affinity, alters intracellular Ca homeostasis, and causes pause-dependent Ca-triggered arrhythmia. *Circulation Research*.

